# Association between exposure to family planning messages on different mass media channels and the utilization of modern contraceptives among young women in Sierra Leone: insights from the 2019 Sierra Leone Demographic Health Survey

**DOI:** 10.1186/s12905-022-01974-w

**Published:** 2022-09-16

**Authors:** Quraish Sserwanja, Patricia Turimumahoro, Lilian Nuwabaine, Kassim Kamara, Milton W. Musaba

**Affiliations:** 1 Programmes Department, GOAL Global, Arkaweet Block 65 House No. 227, Khartoum, Sudan; 2 Uganda Tuberculosis Implementation Research Consortium, Makerere University, Kampala, Uganda; 3 School of Nursing and Midwifery, Aga Khan University, Kampala, Uganda; 4 National Disease Surveillance Programme, Ministry of Health and Sanitation, Freetown, Sierra Leone; 5 Department of Obstetrics and Gynaecology, Mbale Regional Referral and Teaching Hospital, Mbale, Uganda; 6 Department of Obstetrics and Gynaecology, Faculty of Health Sciences, Busitema University, Mbale, Uganda

**Keywords:** Sierra Leone, DHS, Young women, Modern contraceptives, Mass media

## Abstract

**Background:**

Access to sexual and reproductive health information enables young women to make appropriate decisions. We examined the association between exposure to family panning messages on different mass media and the use of modern contraceptives among young women in Sierra Leone.

**Methods:**

This was a secondary analysis of the 2019 Sierra Leone Demographic and Health Survey data of young women aged 15–24 years. Multistage stratified sampling was used to select study participants in the survey. We used multivariable logistic regression to determine the association between exposure to family panning messages on different types mass media channels and utilization of modern contraceptives. All our analyses were done using SPSS version 25.

**Results:**

Out of 6055 young women, 1506 (24.9%, 95% CI 24.0–26.2) were utilizing a modern contraceptive method with the prevalence higher among urban women (26.5%) compared to rural women (23.1%). Less than half (45.6%) had been exposed to family planning messages on mass media (radio 28.6%, television 10.6%, mobile phones 4.2% and newspapers or magazines 2.2%). Young women who had exposure to family planning messages on radio (AOR: 1.26, 95% CI 1.06–1.50) and mobile phones (AOR: 1.84, 95% CI 1.25–2.69) had higher odds of using modern contraceptives compared to their counterparts without the same exposure. Furthermore, having access to internet (AOR: 1.45, 95% CI 1.19–1.78), working (AOR: 1.49, 95% CI 1.27–1.74), being older (20–24 years) (AOR: 1.75, 95% CI 1.46–2.10), being married (AOR: 0.33, 95% CI 0.26–0.42), having visited a health facility within the last 12 months (AOR: 1.34, 95% CI 1.10–1.63), having secondary (AOR: 2.83, 95% CI 2.20–3.64) and tertiary levels of education (AOR: 3.35, 95% CI 1.83–6.13), higher parity (having above one child) AOR: 1.57, 95% CI 1.19–2.08) and residing in the southern (AOR: 2.11, 95% CI 1.61–2.79), northwestern (AOR: 1.87, 95% CI 1.39–2.52), northern (AOR: 2.11, 95% CI 1.59–2.82) and eastern (AOR: 1.68, 95% CI 1.27–2.22) regions of residence were associated with higher odds of modern contraceptives utilization.

**Conclusion:**

In Sierra Leon, only one in four young women were using modern contraception and more than half of them had not had any exposure to family planning messages on the different types of mass media channels. Behavior change communicators can prioritize family planning messages using radio, mobile phones and the internet. In order to publicize and encourage young women to adopt healthy behaviours and increase uptake of modern contraceptive.

**Supplementary Information:**

The online version contains supplementary material available at 10.1186/s12905-022-01974-w.

## Introduction

Family planning remains one of the most cost-effective ways of preventing unplanned pregnancies for many young women across the globe [1]. Correct and consistent use of the available modern contraceptives enables couples to realize the desired birth intervals, ideal family size, and control fertility [2]. Beyond control of fertility, use of modern contraceptives has got several non-contraceptive benefits such as contributing to poverty reduction, increasing gender equity, preventing the spread of HIV (barrier methods), reducing unwanted teenage pregnancies, and lowering infant deaths but also contributes to the progress towards achieving the sustainable development goals (SDGs) [3–5].

Although modern contraceptives are known to be safe and effective, major disparities in uptake/utilisation exist all over the world [6]. For instance, an estimated 214 million women of reproductive-age in developing regions who would want to avoid pregnancy do not use any modern contraceptive method [2]. Much of this unmet need is due to limited access, especially by those in most need such as the adolescents because they tend to have low purchasing power [1, 7]. Despite the early initiation of sexual activity, high fertility rates and high rates of teenage pregnancy in this region, the utilization of modern contraception remains unacceptably low [8–10]. In the same way, governments across the globe strive to improve the uptake of modern contraception, the government of Sierra Leone has committed to increase the contraceptive prevalence rate for modern contraception from the current 20.9 to 33.7% by 2022 [11].

In many developing countries, a lack of knowledge regarding family spacing and contraceptives prevents couples from effectively managing their childbearing [12]. Therefore, use of mass media is one of the strategies that countries across the globe have often employed in the promotion of family planning and to make populations aware of its benefits [13]. Recent studies have proven how effective mass media is in increasing the uptake of these modern contraceptive methods owing to the fact that information can have a positive influence on people’s attitude and actions [13–16]. Mass media such as radio, television, newspapers, billboards, magazines, and digital technologies are vital to the promotion of modern contraception. They are considered a major source of information that can increase the uptake of family planning methods by providing accurate information, building self-efficacy and promoting attitudes, behavioural change and social norms that support the use of modern contraceptives [13, 17]. Media plays a significant role in increasing the awareness and knowledge about contraception, community mobilization and combating misinformation and myths about family methods [8, 18].

Besides being ranked as one of the countries with the worst maternal and child health indicators globally, Sierra Leone is also among the ten countries with the highest rates of teenage pregnancy [19]. Furthermore, an estimated 40% of maternal deaths in Sierra Leone occur among teenagers [20]. In post Ebola and post-conflict context, young women have continued to face profound structural exclusion, poverty, traditional norms related to gender and low literacy levels which factors have negatively affected access to reproductive health services including modern contraceptives utilization [21, 22]. Furthermore, the country’s health system faces frequent stock outs of medical supplies, limited integration of health services, shortage of skilled health workers and poor remuneration of health workers [23–26]. These challenges have contributed to the practice of charging patients including family planning client’s unofficial fees, which is contrary to the official government policy of free reproductive and maternal child health services for all. The most recent service availability and readiness assessment (SARA) survey of health facilities to provide contraception in 10 African countries showed that only 59% of Sierra Leone health facilities providing family planning had family planning guidelines, 75% had at least one staff trained on family planning, 83% had a blood pressure machine and only 42% had all the three items [27].

For the goverment and partners to achieve the set target of improving the contraceptive prevalence rate (CPR) among young women and achieve SDG three, there is need for up todate, and nationally representative information on the factors associated with the utilisation of modern contraeption. In the current study,we examined the association between exposure to family panning messages on different types of mass media and the use of modern contraceptives by young women in Sierra Leone.

## Methods

### Data source and sampling procedure

This was a nationally representative cross-sectional study. We conducted a secondary data analysis of the 2019 Sierra Leone Demographic and Health Survey (SLDHS) data which was conducted from May 2019 to August 2019, to analyze nationally representative key health indicators. Selection of study participants was done through stratified two-stage cluster sampling procedure with 578 enumeration areas (EAs) (214 urban and 364 rural) being selected in the first stage leading to 13,872 households. SLDHS 2019 included women aged 15–49 years who were either permanent residents or slept in the selected household the night before the survey [28]. In this study, we included young women aged 15–24 years. The SLDHS interviewed a weighted sample of 15,574 women in the data set, only 6,0553 were aged 15–24 years. A full protocol with detailed explanation about the data collection process and sampling is available online [28].

### Variables

#### Outcome variables

Utilization of any method of the modern contraceptive method was coded as one (1) while non-utilization was coded as zero (0) [1].

##### Independent variables

Andersen’s behavioral model of health service use was adapted based on the available data and evidence from the literature [1, 4, 15, 18]. According to Andersen’s behavioral model, utilization of healthcare is a function of three major elements: predisposing factors, enabling factors and healthcare needs [29]. The predisposing factors in the model were: age, level of education, region of residence, place of residence, religion, marital status, sex of household head, and parity. Wealth index, working status, internet access, exposure to family planning messages on mass media, being visited by a field health worker, seeking permission and distance to the nearest health facility as an indicator of access were considered as enabling factors, while having visited a health facility within the last 12 months was included in the model as a proxy for the need factor [30].

#### Exposures

Study participants were asked whether if they had heard or read family planning messages on radio, television (TV), newspapers/magazines or mobile phone texts [1]. Responses were recoded as yes and no.

#### Covariates

Table 1 describes the covariates used in this analysis.

**Table 1:** Categorization of independent variables

Variable	Categorization	
Exposure to family planning messages on mass media (phone, television, phone and newspapers/magazines)	Yes and No	Women were asked if they had exposure to family planning mass media messages on each of the four media. Each mass media was asked differently with a yes and no response
Access to internet	Yes and No	This was self-reported with yes for those who reported using internet while no for those who were not using internet
Age	15–19 and 20–24 years	Originally measured as a continuous variable and we categorized it
Residence	Urban and Rural	
Region	Northern, Eastern, Southern, Western and Northwestern	
Religion	Muslims and Christians and others	
Sex of household head	Male and female	
Level of education	No education, primary, secondary and tertiary	Women were asked about the highest level of education ever attended
Working status	Yes and No	All women who were working were categorized as a yes and those that were not working as a no
Wealth index	Richest, richer, middle, poorer and poorest quintiles	Wealth index is a measure of relative household economic status and was calculated by DHS from information on household asset ownership using Principal Component Analysis, which was further categorized into poorest, poorer, middle, richer and richest quintiles
Having visited a health facility within the last 12 months	Yes and No	Women were asked if they had visited a health facility within the last 12 months regardless of the reason with a yes and no response
Having been visited by a field health worker within the last 12 months	Yes and No	Women were asked if they had visited by a field health worker within the last 12 months with a yes and no response
Problems seeking permission and distance to health facility	No big problems and Big problems	SLDHS had three original categories (no problem, no big problem and big problem) however, after data collection, no woman reported no problem
Problems with distance to nearest health facility	No big problems and Big problems	SLDHS had three original categories (no problem, no big problem and big problem) however, after data collection, no woman reported no problem
Parity	None, one and above 1	Was originally a continuous variable that was categorized
Marital status	Married and Not married	Married included both formal and informal unions and not-married included all women that were not in formal or informal unions

### Statistical analysis

In order to account for the unequal probability sampling in different strata [31] and to ensure representativeness of the study results [32], DHS sample weights were applied. SPSS version 25.0 statistical software complex samples package incorporating the following variables in the analysis plan to account for the multistage sample design inherent in the DHS dataset: individual sample weight, sample strata for sampling errors/design, and cluster number was used [33–35]. Tabulation for independent variables was done for proportions and frequencies. Bivariable logistic regression was done to assess the association of each independent variable with utilization of modern contraceptives and crude odds ratio (COR), 95% confidence interval (CI) and *p*-values are presented. Covariates associated with modern contraceptive utilisation with a *p*-value ≤ 0.25 at the bivariable level [36], and not strongly collinear with other independent variables were considered for multivariable logistic regression to assess the independent effect of each variable on modern contraceptive utilisation. Model fitness was assessed with the Hosmer*–*Lemeshow test with a *p*-value of 0.108. Two multivariable logistic regression models were made with the first one having exposure to family planning mass media message variables and the final one including other independent variables. All variables in the model were assessed for collinearity, which was considered present if the variables had a variance inflation factor (VIF) greater than 3. However, no VIF above 2.5 was reported. Adjusted odds ratios (AOR), 95% Confidence Intervals (CI) and *p*-values were calculated with statistical significance level set at *p*-value < 0.05. Sensitivity analysis was conducted with rural–urban stratification.

## Results

A total of 6055 young women were included in the analysis. Of these, 1506 (24.9%, 95% CI 24.0–26.2) were utilizing modern contraceptives. Exposure to family planning messages was highest through radio (28.6%) followed by TV (10.6%), phone (4.2%) and least by newspapers or magazines (2.2%). Majority of the young women had secondary education (62.9%), were not married (69.6%), not working (53.4%), resided in urban areas (52.9%), aged 15–19 years (56.6%), resided in the Western region (26.5%) and were Muslims (75.8%). Details are in Table 2.

**Table 2:** Socio-demographic characteristics of young women in Sierra Leone as per the 2019 SLDHS

Characteristics	N = 6055	%
*Heard family planning messages on radio*		
No	4322	71.4
Yes	1733	28.6
*Heard family planning messages on TV*		
No	5411	89.4
Yes	644	10.6
*Read family planning messages in newspapers/magazines*		
No	5922	97.8
Yes	133	2.2
*Read family planning messages on phone*		
No	5799	95.8
Yes	256	4.2
*Access to internet*		
No	4990	82.4
Yes	1065	17.6
*Age*		
15–19	3427	56.6
20–24	2628	43.4
*Residence*		
Urban	3201	52.9
Rural	2854	47.1
*Region*		
Western	1607	26.5
Eastern	1139	18.8
Northwestern	1010	16.7
Northern	1244	20.6
Southern	1054	17.4
*Religion*		
Islam	4591	75.8
Christianity and others	1464	24.2
*Sex household head*		
Male	4065	67.1
Female	1990	32.9
*Working status*		
Not working	3232	53.4
Working	2823	46.6
*Education level*		
No education	1121	18.5
Primary education	975	16.1
Secondary education	3810	62.9
Tertiary	150	2.5
*Wealth index*		
Poorest	799	13.2
Poorer	967	16.0
Middle	1148	19.0
Richer	1552	25.6
Richest	1591	26.3
*Visited health facility within 12 months*		
No	3277	54.1
Yes	2778	45.9
*Visited by field health worker*		
No	4581	75.6
Yes	1474	24.4
*Permission to access healthcare*		
Big problem	1429	23.6
Not big problem	4626	76.4
*Distance to health facility*		
Big problem	2449	40.4
Not big problem	3606	59.6
*Marital status*		
Not married	4213	69.6
Married	1842	30.4
*Parity*		
0	3667	60.6
1	1468	24.2
Above 1	920	15.2
*Modern contraceptive use*		
Yes	1506	24.9 (95% CI 24.0–26.2)
No	4549	75.1

### Association between exposure to family planning mass media messages and modern contraceptive utilization

After adjusting for other variables, factors that were statistically associated with modern contraceptives utilization were hearing messages on radio (AOR: 1.26, 95% CI 1.06–1.50) and reading texts on mobile phones (AOR: 1.84, 95% CI 1.25–2.69). Other covariates that were significant included being a working woman (AOR: 1.49, 95% CI 1.27–1.74), being older (20–24 years) (AOR: 1.75, 95% CI 1.46–2.10), being married (AOR: 0.33, 95% CI 0.26–0.42), having visited a health facility within the last 12 months (AOR: 1.34, 95% CI 1.10–1.63), having access to internet (AOR: 1.45, 95% CI 1.19–1.78), secondary (AOR: 2.83, 95% CI 2.20–3.64) and tertiary level of education (AOR: 3.35, 95% CI 1.83–6.13), higher parity (having one child AOR: 1.34, 95% CI 1.09–1.65 and having above one child AOR: 1.57, 95% CI 1.19–2.08) and residing in the southern (AOR: 2.11, 95% CI 1.61–2.79), northwestern (AOR: 1.87, 95% CI 1.39–2.52), northern (AOR: 2.11, 95% CI 1.59–2.82) and eastern (AOR: 1.68, 95% CI 1.27–2.22) regions of residence. Details are in Table 3.

**Table 3:** Exposure to family planning messages and utilization of modern contraceptives among young women in Sierra Leone

Characteristics	Crude model cOR (95% CI)	*p*-value	Adjusted model I aOR (95% CI)	*p*-value	Adjusted model II aOR (95% CI)	*p*-value
*Heard messages on radio*		** < 0.001**		** < 0.001**		**0.008**
No	**1**		**1**		**1**	
Yes	**1.63 (1.38–1.91)**		**1.56 (1.33–1.84)**		**1.26 (1.06–1.50)**	
*Heard messages on TV*		0.098		0.127		0.333
No	**1**		1		1	
Yes	**1.26 (0.96–1.65)**		0.79 (0.59–1.07)		0.86 (0.63–1.17)	
*Messages in newspapers*		0.114		0.611		0.536
No	**1**		1		1	
Yes	1.50 (0.91–2.49)		0.86 (0.48–1.53)		0.83 (0.46–1.50)	
*Read messages on phone*		** < 0.001**		** < 0.001**		
No	**1**		**1**		**1**	**0.002**
Yes	**2.63 (1.90–3.65)**		**2.34 (1.64–3.35)**		**1.84 (1.25–2.69)**	
*Access to internet*		** < 0.001**				
No	**1**				**1**	** < 0.001**
Yes	**1.88 (1.57–2.24)**				**1.45 (1.19–1.78)**	
Age		** < 0.001**				** < 0.001**
15–9	**1**				**1**	
20–24	**1.60 (1.39–1.85)**				**1.75 (1.46–2.10)**	
*Residence*		**0.025**				0.082
Rural	**1**				1	
Urban	**1.20 (1.02–1.41)**				1.26 (0.97–1.63)	
*Region*						
Western	1				1	
Southern	**1.33 (1.05–1.69)**	**0.018**			**2.11 (1.61–2.79)**	** < 0.001**
Northwestern	1.19 (0.92–1.54)	0.194			**1.87 (1.39–2.52)**	** < 0.001**
Northern	**1.53 (1.19–1.97)**	**0.001**			**2.11 (1.59–2.82)**	** < 0.001**
Eastern	**1.27 (1.01–1.59)**	**0.039**			**1.68 (1.27–2.22)**	** < 0.001**
*Religion*		**0.001**				0.221
Islam	**1**				1	
Christianity and others	**1.31 (1.12–1.52)**				1.11 (0.94–1.30)	
*Sex household head*		**0.001**				0.732
Male	**1**				1	
Female	**1.29 (1.11–1.51)**				1.03 (0.87–1.22)	
*Working status*		**0.008**				** < 0.001**
Not working	**1**				**1**	
Working	**1.20 (1.05–1.38)**				**1.49 (1.27–1.74)**	
*Education level*						
No education	1				1	
Primary education	1.18 (0.87–1.59)	0.288			1.19 (0.87–1.62)	0.280
Secondary education	**2.89 (2.34–3.57)**	** < 0.001**			**2.83 (2.20–3.64)**	** < 0.001**
Tertiary	**4.18 (2.55–6.84)**	** < 0.001**			**3.35 (1.83–6.13)**	** < 0.001**
*Wealth index*						
Poorest	1				1	
Poorer	1.14 (0.82–1.57)	0.436			1.07 (0.78–1.47)	0.659
Middle	**1.51 (1.13–2.03)**	**0.006**			1.15 (0.87–1.51)	0.320
Richer	**1.67 (1.24–2.24)**	**0.001**			0.95 (0.66–1.35)	0.757
Richest	1.18 (0.87–1.60)	0.295			0.71 (0.47–1.08)	0.108
*Facility visit within 12 months*		** < 0.001**				**0.004**
No	**1**				**1**	
Yes	**1.41 (1.22–1.64)**				**1.34 (1.10–1.63)**	
*Visited by field health worker*		** < 0.001**				0.150
No	**1**				1	
Yes	**1.43 (1.20–1.70)**				1.16 (0.95–1.41)	
*Permission to access healthcare*		**0.033**				0.939
Big problem	**1**				1	
Not big problem	**1.22 (1.02–1.47)**				1.01 (0.82–1.25)	
*Distance to health facility*		**0.005**				0.077
Big problem	**1**				1	
Not big problem	**1.26 (1.07–1.48)**				1.19 (0.98–1.45)	
*Marital status*		** < 0.001**				** < 0.001**
Not married	**1**				**1**	
Married	**0.53 (0.45–0.63)**				**0.33 (0.26–0.42)**	
*Parity*						
0	**1**					
1	**1.21(1.02–1.43)**	**0.025**			**1.34 (1.09–1.65)**	**0.005**
Above 1	1.02(0.85–1.25)	0.799			**1.57 (1.19–2.08)**	**0.001**

Bold: significant at *p*-value < 0.05

#### Sensitivity analysis

After stratifying the data by residence, the prevalence of modern contraceptive utilization was higher among urban women (26.5%) compared to their rural counterparts (23.1%). Regarding exposure to mass media, exposure to radio messages was the highest in both groups (rural 21.1% and urban 35.4%) while exposure to newspaper messages was the lowest (rural 0.5% and urban 3.7%). Only 2.2% of rural women owned mobile phones compared to 6% among urban women.

In the multivariable analysis, among the main exposure, exposure to family planning messages on phone was the only mass media variable that was associated with higher odds of modern contraception among urban women (aOR: 1.76, 95% CI 1.14–2.73) while exposure to family planning messages on phone (aOR: 2.62, 95% CI 1.26–5.43) and on radio (aOR: 1.43, 95% CI 1.09–1.87) were associated with higher odds of modern contraception among rural women.

Among the covariates, only higher parity and higher education were associated with higher odds of modern contraceptive utilization among both rural and urban women while working, having visited a health facility, having access to internet, being older and belonging to all the other regions except the western region were associated with higher odds among urban women. Among rural women, having been visited by a field health worker and belonging to the richest quintile were associated with higher odds of modern contraceptive utilization while being married was associated with less odds of modern contraception as shown in Additional file 1.

## Discussion

Our findings indicate that 24.9% of young women were utilizing modern contraceptives with the prevalence being higher among urban (26.5%) compared to rural (23.1%) women. Less than half of them had been exposed to family planning messages through mass media and the readily available types of media in descending order were radio, TV, phone messages, and print media such as newspapers and magazines.

The low exposure to FP messaging on traditional media reveals a need to increase the reach of modern contraceptive mass media messaging. As well as the need to create targeted messages for key populations to whom traditional mass media messaging may not be readily accessible and available such as younger unmarried women and the women of lower educational levels. With an estimated fertility rate of 4.2 births per woman [28], effort must be placed on mass media messaging that not only influences behavioral change in key underserved populations but also invest in extending the reach of current accessible media platforms in order to achieve the SDG 3.7.1 target of universal access to modern contraception by 2030.

We found that young women who had exposure to family planning messages on radio and mobile phones had higher odds of using modern contraceptives compared to their counterparts without the same exposure. Most people depend on listening to radio as the primary source of information especially in rural areas of Africa [37]. According to the 2019 SLDHS, radio remains the commonest mass media women are exposed to [28]. Not only is radio widespread and popular, it’s also a cost-effective way to share information with a large audience, capable of delivering information quickly and interactive where the audience is able to give feedback [37]. The positive association between mobile phone messaging and utilization of modern contraception is in agreement with similar reports from similar low resource settings [38, 39]. The increasing penetration of mobile phones or digital media platforms are an emerging opportunity to engage young audiences in most need [39]. Mobile phones also represent the most rapid expansion as a medium for mass media messaging globally and in Sierra Leone [40].

Overall, mass media messaging leads to positive healthy behavioral change through regular, frequent and targeted broadcasted programs and announcements that inform young women about the benefits of accessing reproductive health services including contraception [41]. They also inform the masses about the availability and working hours of public health facilities that provide free services. In addition to being relatively low cost per person exposed, traditional mass media further enables an easily augmentable frequency of delivery and ability to have high control over content [42]

The DHS did not provide data on messaging through use of internet but reported internet access and in this study, young women who had access to internet had higher odds of modern contraceptive utilization compared to those without internet access. While internet access, use of social media platforms and evidence to support use of mobile health technology is still limited in Sierra Leone, its utilization is expected to exponentially increase over the next decade [43]. Different internet resources such as web pages, social media platforms, bulletin boards, and chatrooms enables access to information in real time for a potentially large number of young women [44, 45]. Internet enables young women to easily access information about their sexuality with a high degree of interactivity, anonymity and confidentiality [45]. Besides being a source of health information that empowers young women to have higher contraceptive literacy, internet can as well be used to purchase contraceptives [46, 47] and also to provide easy to understand information on how to deal with contraceptives’ side effects which are a major reason for low uptake and discontinuation of modern contraceptives [48, 49].

Several sociodemographic factors were associated with utilization of modern contraceptive and these included; age, region, level of education, working, marital status, visit to the health facility in the last 12 months were significantly associated with utilization of modern contraceptives which is supported by most of the literature [1, 50–52].

## Strengths and limitations

This analysis is based on the current 2019 DHS data that is nationally representative and was weighted to facilitate generalizability to all women of reproductive age (15–49 years) in Sierra Leone. This secondary analysis from the DHS is based on data generated from standardized training and validation procedures endorsing its internal and external validity. These findings should be interpreted taking into account that this was an observational cross-sectional analysis of correlation which inherently does not establish temporality. We also anticipate that there could have been response bias such as recall bias during the process of data collection as data collection is retrospective and some questioning formats may lead to social desirability bias.

## Conclusion

In Sierra Leone, only one in four women are exposed to modern contraceptive through traditional mass media messaging channels available in Sierra Leone. This study suggests that radio, phone text messaging and internet could increase exposure and subsequently positively impact utilization of modern contraceptives. To ensure effective use of mass media messages and that correct and easy to understand information is passed on, we would like to recommend the need for sexual and reproductive health workers and behavioral change communicators to be highly involved in the designing and dissemination of these messages. Policies and programmes aimed at enabling access to affordable phones and internet to young women need to be considered. Furthermore, reproductive health stakeholders need to ensure context specific policies and programmes for rural and urban women having shown that exposure to phone family planning messages and use of internet being significant in urban areas while exposure to family planning messages on radio and phone were significant in rural areas.

Besides mass media exposure, visiting a health facility was associated with higher odds of contraceptive utilization hence there is need to further strengthen integration of health services and ensure that family planning counselling is provided to all young women accessing health facilities especially the married, young, less educated, those with no children and those from the western region.

## Supplementary Information

**Additional file 1.** Rural-Urban stratification of exposure to family planning messages and utilization of modern contraceptives among young women in Sierra Leone.

## Data Availability

The data set used is openly available upon permission from MEASURE DHS website (URL: https://www.dhsprogram.com/data/available-datasets.cfm).

## Abbreviations

EA: Enumeration area
AOR: Adjusted odds ratio
CI: Confidence interval
COR: Crude odds ratio
DHS: Demographic Health Survey
SLDHS: Sierra Leone Demographic Health Survey
OR: Odds ratio
SD: Standard deviation
WHO: World Health Organization
SPSS: Statistical package for social science
TV: Television
