# Supplementary material for: Association between exposure to family planning messages on different mass media channels and the utilization of modern contraceptives among young women in Sierra Leone: insights from the 2019 Sierra Leone Demographic Health Survey
Source: BMC Womens Health. 2022 Sep 16;22:376. doi: 10.1186/s12905-022-01974-w (PMC9479264; doi:10.1186/s12905-022-01974-w)
Supplement: Supplementary file 1 — Additional file 1. Rural-Urban stratification of exposure to family planning messages and utilization of modern contraceptives among young women in Sierra Leone. [file 12905_2022_1974_MOESM1_ESM.docx]

**Rural-Urban stratification of exposure to family planning messages and utilization of modern contraceptives among young women in Sierra Leone**

| **Characteristics** | **Rural**  **Crude model**  **cOR (95% CI)** | **Rural**  **Adjusted model**  **aOR (95% CI)** | **Urban**  **Crude model**  **cOR (95% CI)** | **Urban**  **Adjusted model**  **aOR (95% CI)** |
| --- | --- | --- | --- | --- |
| **Heard Messages on Radio** |  |  |  |  |
| No | **1** | **1** | **1** | 1 |
| Yes | **2.26 (1.76-2.90)** | **1.43 (1.09-1.87)** | **1.27 (1.02-1.57)** | 1.10 (0.88-1.38) |
| **Heard messages on TV** |  |  |  |  |
| No | **1** | 1 | 1 | 1 |
| Yes | **3.08 (1.59-5.97)** | 1.53 (0.85-2.76) | 1.05 (0.78-1.42) | 0.90 (0.64-1.27) |
| **Messages in newspapers** |  |  |  |  |
| No | **1** | 1 | 1 | 1 |
| Yes | 1.01 (0.28-3.65) | 0.23 (0.03-1.68) | 1.47 (0.85-2.55) | 1.05 (0.56-1.97) |
| **Read messages on phone** |  |  |  |  |
| No | **1** | **1** | **1** | **1** |
| Yes | **4.30 (2.18-8.47)** | **2.62 (1.26-5.43)** | **2.13 (1.46-3.09)** | **1.76 (1.14-2.73)** |
| **Access to internet** |  |  |  |  |
| No | **1** | 1 | **1** | **1** |
| Yes | **3.04 (1.89-4.88)** | 1.47 (0.90-2.38) | **1.66 (1.36-2.03)** | **1.58 (1.26-1.98)** |
| **Age** |  |  |  |  |
| 15 to 19 | 1 |  | **1** | **1** |
| 20 to 24 | 1.08 (0.88-1.34) |  | **2.22 (1.83-2.68)** | **1.94 (1.52-2.48)** |
| **Region** |  |  |  |  |
| Western | 1 | 1 | **1** | 1 |
| Southern | 0.60 (0.25-1.47) | 0.88 (0.26-2.93) | **1.91 (1.44-2.55)** | **2.53 (1.85-3.46)** |
| Northwestern | 0.51 (0.20-1.27)* | 0.88 (0.26-2.98) | **1.95 (1.45-2.64)** | **2.13 (1.53-2.99)** |
| Northern | 0.80 (0.33-1.99) | 1.05 (0.32-3.45) | **1.69 (1.24-2.30)** | **1.88 (1.35-2.63)** |
| Eastern | 0.50 (0.21-1.24)* | 0.68 (0.21-2.24) | **1.70 (1.31-2.20)** | **1.93 (1.38-2.71)** |
| **Religion** |  |  |  |  |
| Islam | **1** | **1** | 1 |  |
| Christianity and others | **1.61 (1.27-2.04)** | **1.50 (1.16-1.92)** | 1.10 (0.90-1.33) |  |
| **Sex household head** |  |  |  |  |
| Male | 1 |  | **1** | 1 |
| Female | 1.12 (0.89-1.41) |  | **1.36 (1.11-1.66)** | 1.16 (0.93-1.43) |
| **Working status** |  |  |  |  |
| Not working | 1 |  | **1** | **1** |
| Working | 0.95 (0.78-1.15) |  | **1.68 (1.39-2.03)** | **1.58 (1.27-1.98)** |
| **Education Level** |  |  |  |  |
| No Education | 1 | 1 | 1 | 1 |
| Primary Education | 1.40 (1.00-1.97)* | 1.14 (0.79-1.63) | 0.79 (0.44-1.42) | 0.86 (0.47-1.58) |
| Secondary Education | **4.59 (3.57-5.88)** | **3.30 (2.52-4.33)** | **1.51 (1.04-2.21)** | **1.62 (1.04-2.53)** |
| Tertiary | **6.02(1.42-25.60** | 3.17 (0.74-13.54) | **2.47 (1.34-4.54)** | 2.05 (0.98-4.27) |
| **Wealth Index** |  |  |  |  |
| Poorest | 1 | 1 | 1 | 1 |
| Poorer | 1.12 (0.80-1.55) | 0.99 (0.72-1.38) | 0.60 (0.12-2.92) | 0.77 (0.20-2.99) |
| Middle | **1.47 (1.08-1.99)** | 1.02 (0.78-1.34) | 0.60 (0.12-2.90) | 0.81 (0.21-3.19) |
| Richer | 1.52 (0.97-2.39)* | 0.61 (0.37-1.01) | 0.58 (0.13-2.55) | 0.79 (0.22-2.88) |
| Richest | 1.39 (0.63-3.06) | 0.46 (0.22-0.98) | 0.40 (0.09-1.78)* | 0.63 (0.17-2.35) |
| **Facility visit within 12 months** |  |  |  |  |
| No | 1 |  | **1** | **1** |
| Yes | 1.12 (0.90-1.39) |  | **1.79 (1.45-2.22)** | **1.44 (1.09-1.90)** |
| **Visited by field health worker** |  |  |  |  |
| No | **1** | **1** | 1 | 1 |
| Yes | **1.64 (1.30-2.07)** | **1.46 (1.11-1.91)** | 1.30 (1.00-1.68)* | 0.99 (0.75-1.31) |
| **Permission to access healthcare** |  |  |  |  |
| Big problem | **1** | 1 | 1 |  |
| Not big problem | **1.36 (1.04-1.78)** | 1.07 (0.82-1.40) | 1.00 (0.77-1.31) |  |
| **Distance to health facility** |  |  |  |  |
| Big problem | **1** | 1 | 1 |  |
| Not big problem | **1.44 (1.12-1.84)** | 1.20 (0.93-1.55) | 1.02 (0.80-1.30) |  |
| **Marital status** |  |  |  |  |
| Not married | **1** | **1** | 1 | **1** |
| Married | **0.37 (0.30-0.45)** | **0.34 (0.26-0.46)** | 0.84 (0.63-1.10)* | **0.42 (0.29-0.61)** |
| **Parity** |  |  |  |  |
| 0 | 1 | **1** | **1** | **1** |
| 1 | 0.80 (0.62-1.03)* | **1.33 (1.02-1.75)** | **1.76 (1.41-2.20)** | **1.56 (1.18-2.07)** |
| Above 1 | 0.79 (0.61-1.03)* | **2.15 (1.52-3.04)** | **1.51 (1.11-2.05)** | **1.72 (1.13-2.61)** |

**Bold**: significant at p-value < 0.05, *P-value > 0.05 and <0.25
